# Our experience diagnosing 225 patients with cervical glandular lesions: current technologies, lessons learned, and areas for improvement

**DOI:** 10.1186/s13000-023-01428-3

**Published:** 2024-01-26

**Authors:** Yan Qin, Junyi Deng, Yuexian Ling, Tao Chen, Hongyi Gao

**Affiliations:** 1 Department of Pathology, Guangdong Women and Children Hospital, Guangzhou, 511400 China; 2 Yangjiang Key Laboratory of Respiratory Disease, People’s Hospital of Yangjiang, Yangjiang, Guangdong 529500 China; 3 The First Affiliated Hospital of Guangzhou Medical University, Guangzhou, 510120 China; 4 Guangdong Women and Children Hospital, No. 521, Xingnan Avenue, Panyu District, Guangzhou, China

**Keywords:** Adenocarcinoma *in situ*, AIS, Cervical adenocarcinoma, ADC, Cervical cancer, Papanicolaou test, HPV

## Abstract

**Objective:**

To explore the relative sensitivity of different methods for detecting cervical glandular lesions.

**Methods:**

A total of 225 patients with cervical glandular lesions diagnosed from January 2018 to February 2023 were retrieved from the pathology database of Guangdong Maternal and Child Health Hospital, and their clinicopathological features were reviewed.

**Results:**

Four human papillomavirus (HPV) genotypes: HPV18, 16, 45, and 52, dominated all glandular lesions, and accounting for 74.10% of HPV-positive tumors. Furthermore, 36.89% of abnormal squamous cells were diagnosed as abnormal based on cytological examinations leading to the detection of cervical glandular lesions; only 16.89% were diagnosed based on the initial detection of abnormal glandular cytology. The most common abnormal cervical screening result was ASC-US on cytology (14.22%), followed by HSIL (11.56%). Only few number of patients were diagnosed with or suspected of having cervical adenopathy via a Pap test (18.22%). Nearly one-third of cervical glandular lesions cases were not detected on the Pap test; but were diagnosed upon cervical biopsy or based on the histological examination of ECC, LEEP, or CKC specimens. The LEEP or CKC biopsy specimens had negative margins in 49 cases (40.83%), while the margins were positive in the other 71 cases (59.17%). Five cases (10.20%) with negative margins still had residual lesions following total hysterectomy, and 19 (26.76%) with positive margins had no residual lesions after total hysterectomy.

**Conclusion:**

The ability to detect cervical glandular lesions varies for routine HPV genotyping, Pap test, or biopsy/ECC, with different sensitivities and advantages and disadvantages for each method.

## Introduction

Cervical cancer is a major malignant tumor that endangers the health and lives of women. As the second most prevalent cancer after breast cancer, the global incidence of cervical cancer exceeds 500,000 cases per year. In recent decades, cervical squamous cell carcinoma incidence has declined steadily with the advancement in the system for preventing and treating cervical cancer. However, the cervical adenocarcinoma (ADC) incidence rate continues to rise.

Adenocarcinoma is the second most common histological type of cervical cancer, with an upward trend in incidence rate, accounting for 20 ~ 25% of cervical cancer [1–4]. Moreover, the prognosis for cervical squamous cell carcinoma was poorer than that for ADC diagnosed over the same period [4]. It is evident that persistent high-risk human papillomavirus (hr-HPV) infection is the leading cause of cervical cancer.

Since all the methods, including HPV genotyping, Pap test, cervical biopsy, endocervical curettage (ECC), loop electrosurgical excision procedure (LEEP), or cold knife conization (CKC), and total hysterectomy, can be used for preliminary diagnosis of cervical glandular lesions, it became more and more essential to determine the relative sensitivity of different diagnostic methods. Therefore, we started this retrospective study to analyze the cervical glandular lesions cases that diagnosed at our hospital, the pathology database of Guangdong Maternal and Child Health Hospital.

A total of 225 patients with cervical glandular lesions diagnosed from January 2018 to February 2023 were retrieved from the pathology database, and their clinicopathological features were reviewed. Our results indicated that the ability to detect cervical glandular lesions varies for routine HPV genotyping, Pap test, or biopsy/ECC, with different sensitivities and advantages and disadvantages for each method; though further standardization is needed for cervical cancer screening in China.

## Materials and methods

This retrospective statistical analysis was approved by the Research Ethics Committee of Guangdong Maternal and Child Health Hospital in China. We conducted a retrospective statistical analysis on women diagnosed and treated for cervical glandular lesions from January 2018 to February 2023. Furthermore, data resources were reviewed, such as medical records of outpatient and inpatient gynecological and obstetric colposcopy clinics and the pathological database of Guangdong Maternal and Child Health Hospital. The following characteristics of patients were studied: age, sex, HPV genotyping, Pap test results, cervical biopsy, ECC results, LEEP or CKC biopsy, and pathological results of total hysterectomy specimens. Women with a pathological diagnosis of cervical adenopathy obtained via cervical biopsy, LEEP, or CKC diagnostic resection met the inclusion criteria, while exceptions were as follows, pregnant women or women with a history of gynecological cancer, non-HPV related ADC (such as gastric, clear cell, mesonephric, and endometrioid adenocarcinomas).

The HybridMax technology was used to detect 21 subtypes of HPV. The DNA extraction kit and HPV gene microarray typing test kit were provided by the Hong Kong Kepu Company, which obtained a total of 21 types of HPV:14 high-risk types (16, 18, 31, 33, 35, 39, 45, 51, 52, 56, 58, 59, 66, and 68), five low-risk types (6, 11, 42, 43, and 44), and two other types (53 and CP8304). A total of 14 types of HPV were identified as positive for high-risk HPV. Some of the consultation case data came from external hospitals; thus, the specific methods and classification were unknown.

Cervical thin-layer liquid-based cytology mainly uses HOLOGY TCT and Chinese Epson LCT products. Moreover, the 2014 Bethesda Cervical Cytology Reporting System (TBS) was adopted to interpret cytological results.

Histological feature evaluation: two or more gynecological pathology experts microscopically examined the pathological slides stained with hematoxylin and eosin (H&E).

### Statistical analysis

Statistical analyses were performed using SPSS 21.0 version. Descriptive data were expressed as percentages/ranges. Statistical analyses were conducted using the chi-squared test and Fisher’s exact test. A *P*-value less than 0.05 was considered statistically significant.

## Results

### HPV test results

Among 225 patients with cervical glandular lesions, 19 cases (8.44%) of cervical adenocarcinoma in situ (AIS) were found, and 71 cases (31.56%) of ADC were found. There were 135 cases (60%) of mixed glandular and squamous lesions (including AIS and ADC) combined with squamous intraepithelial lesions (including low-grade squamous intraepithelial lesions (LSIL)/high-grade squamous intraepithelial lesions (HSIL)/squamous cell carcinoma (SCC)). Amongst the 19 AIS patients, 17 (89.45%) had HPV16 and/or 18 infections; three cases (15.79%) were each infected with HPV52 and 58, while two cases (10.53%) had unknown HPV results. Furthermore, there were 15 cases of single HPV infection (88.24%) and two cases of multiple mixed HPV infections (11.76%). Of the 71 ADC patients, 50 (70.42%) had HPV16 and/or 18 infections, while HPV infection was detected in seven (9.86%) cases. Among the 50 patients there were 43 cases (82.69%) of single HPV infection and nine cases (17.31%) of multiple mixed HPV infections. Among the 135 cases of mixed glandular and squamous lesions, 98 cases (72.59%) had HPV16 and/or 18 infections, 11 cases (8.15%) had HPV45 infection, and 10 cases (7.41%) did not have a detectable HPV infection. Additionally, there were 89 (80.91%) with single HPV infections and 21 (19.01%) with multiple mixed HPV infections.

Concludingly, four HPV genotypes (HPV18, 16, 45, and 52) predominated in all types of glandular lesions, accounting for 74.10% of HPV-positive tumors. Among the 88.37% of lesions that were HPV- positive, HPV18 (36.25%) was the most common, followed by HPV16 (29.48%), HPV45 (4.75%), and HPV52 (3.5%). Other high-risk HPVs(hr HPVs), including HPV31, 33, 51, 58, 59, 66, 68, 53 and CP8304, were less common, while high-risk types 35and 39 were not detected. Furthermore, the majority (82.12%) of hrHPV-positive tumors showed the presence of a single virus type, while 17.88% of hrHPV- positive tumors had multiple virus types detected. Overall, there was no HPV detected in 6.77% of tumors; another 11.55% of tumors had an unknown status (Tables 1 and 2).

**Table 1:** Distribution of hrHPV genotypes

HPV types	AIS	ADC	MASL	Total (%)
16	7	23	44	74(29.48%)
18	10	27	54	91(36.25%)
31	0	0	1	1(0.40%)
33	0	4	2	6(2.39%)
45	0	1	11	12(4.78%)
51	0	0	3	3(1.20%)
52	2	1	6	9(3.59%)
58	1	0	0	1(0.40%)
59	0	1	1	2(0.80%)
62	0	0	1	1(0.40%)
66	0	0	1	1(0.40%)
68	0	0	1	1(0.40%)
hr-HPV	0	1	2	3(1.20%)
Not detected	0	7	10	17(6.77%)
Unknown	2	12	15	29(11.55%)

Abbreviations: AIS, adenocarcinoma in situ; ADC, cervical adenocarcinoma;

MASL, mixed glandular and squamous lesions; hr-HPV, high-risk HPV (infection rate)

**Table 2:** Proportion of single and mixed multiple hrHPV infection cases

Histopathological diagnosis	HPV-positive cases	Single hrHPV types	Multiple hrHPV types
AIS(n = 19)	17(89.47%)	15(88.24%)	2(11.76%)
Ada(n = 71)	52(73.24%)	43(82.69%)	9(17.31%)
MASL(n = 135)	110(81.48%)	89(80.91%)	21(19.01%)
	179(79.56%)	147(82.12%)	32(17.88%)

### The bethesda system (TBS) interpretation results

From 19 AIS patients, only 11 (57.89%) showed abnormalities in the Pap test, while seven cases (36.84%) did not have any detectable lesions based on the Pap test. Among the 71 cases of ADC, 32 (45.07%) showed abnormalities in the Pap test, while 22 cases (30.99%) did not show any lesions. Among 135 cases of mixed glandular and squamous lesions, 84 cases (62.22%) presented abnormalities in the Pap test, while 32 cases (23.70%) did not show any lesions. Based on a comprehensive analysis, 36.89% (n = 83) of cytological abnormalities leading to the detection of cervical glandular lesions were diagnosed as squamous cell abnormalities. Only 16.89% (n = 38) of the diagnoses were prompted by the detection of abnormal glandular cytology. The most common abnormal result of cervical screening was ASC-US based on a cytological examination (n = 32, 14.22%), followed by HSI (n = 26, 11.56%). Only a few patients were diagnosed with or suspected of having cervical adenomatous lesions based on a Pap test (n = 41, 18.22%). Those detected to have abnormal glandular cytology were classified as AGC-NOS (n = 6, 2.66%), AGC (n = 19, 8.44%), AGC-FN (n = 12, 5.33%), AIS (n = 1, 0.44%), and ADC (n = 3, 1.33%).

A cytological examination revealed that there were 61 women (27.11%) with NILM. Nearly one-third of cases of cervical glandular lesions did not have any issues observed in the Pap test, and the majority were diagnosed through cervical biopsy or cervical curettage, LEEP or based on the CKC specimen histology (Tables 3 and 4).

**Table 3:** Pap test findings in patients

AIS (n = 19)		ADC (n = 71)		MASL (n = 135)	
Cytology	N(%)	Cytology	N(%)	Cytology	N(%)
AGC-NOS	1(5.26%)	AGC-NOS	1(1.41%)	AGC-NOS	4(2.96%)
AGC	3(15.79%)	AGC	4(5.63%)	AGC	6(4.44%)
AGC-FN	1(5.26%)	AGC-FN	5(7.04%)	AGC-FN	6(4.44%)
ASC-US/LSIL	4(21.05%)	AIS	1(1.41%)	AGC + HSIL(Suspicious for SCC)	2(1.48%)
ASC-H/HSIL	2(10.53%)	AGC + ASC-H	1(1.41%)	AGC + ASC-H	1(0.74%)
NILM	7(36.84%)	AGC + ASC-US	1(1.41%)	AGC + ASC-US	1(0.74%)
Not done or Unknown	1(5.26%)	ASC-US/LSIL	10(14.08%%)	ASC-US /LSIL	31(22.96%)
		ASC-H/ASC-H/SCC	7(9.86%)	ASC-H/HSIL/SCC	25(18.52%)
		AGC + ASC-H	1(1.41%)	LSIL-H	4(2.96%)
		Malignancy	1(1.41%)	HSIL, Suspicious for SCC	2(1.48%)
		NILM	22(30.99%)	ADC	2(1.48%)
		Not done or Unknown	17(23.94%)	NILM	32(23.7%)
				Not done or Unknown	19(14.07%)

Abbreviations: AGC-NOS, atypical glandular cells, not otherwise specified; AGC, atypical glandular cells; AGC-FN, atypical glandular cells-favor neoplastic; AIS, adenocarcinoma in situ; ADC, cervical adenocarcinoma; ASC-US, atypical squamous cell of undetermined significance; ASC-H, atypical squamous cells, cannot rule out high-grade squamous intraepithelial lesion; SCC, squamous cell carcinoma; HSIL, high-grade squamous intraepithelial lesion; LSIL, low-grade squamous intraepithelial lesion; NILM, negative for intraepithelial lesion or malignancy

**Table 4:** Comparison of Pap test cytology, biopsy, and/or ECC and cervical conization in the diagnosis of endocervical glandular lesions

Diagnostic modality	AIS	ADC	MASL	Total(Sensitivity%)
Pap test	0(0.00%)	1(1.41%)	2(1.48%)	3(1.33%)
Biopsy/ECC	7(36.84%)	36(50.7%)	14(10.37%)	57(25.33%)
Combined cytology and biopsy/ECC	11(57.89%)	32(45.01%)	59(43.70%)	102(45.33%)
Conization	1(5.26%)	2(2.82%)	60(44.44%)	63(28.00%)

Abbreviations: ECC, endocervical curettage; Pap, Papanicolaou

### Pathological biopsy analysis

Among 135 cases of mixed glandular and squamous lesions, 29 cases (21.48%) of AIS were combined with HSIL and LSIL. Eight cases (5.93%) of ADC combined with HSIL and LSIL simultaneously. 25 cases (18.52%) and 10 cases (7.40%) of AIS combined with HSIL and LSIL simultaneously. Two cases (1.48%) simultaneously merged SCC, 31 cases (22.96%), and 20 cases (14.81%) simultaneously merged HSIL and LSIL with ADC, respectively 10 cases (7.40%) of ADC combined with SCC (Table 5).

**Table 5:** Histological types of mixed glandular and squamous lesions in 135 cases

Histological type	Total(%)
AIS + HSIL + LSIL	29(21.48%)
AIS + HSIL	25(18.52%)
AIS + LSIL	10(7.40%)
AIS + SCC	2(1.48%)
ADC + HSIL + LSIL	8(5.93%)
ADC + HSIL	31(22.96%)
ADC + LSIL	20(14.81%)
ADC + SCC	10(7.40%)

All 19 AIS cases underwent cervical biopsy and/or ECC; 15 cases (78.95%) underwent LEEP or CKC biopsy and 17 (89.47%) underwent a total hysterectomy. Of the 15 patients who underwent a LEEP or CKC, 11 (73.33%) had negative margins and four (26.67%) had positive margins. Moreover, among the 11 cases with negative margins in LEEP or CKC, one case (9.09%) had a small amount of residual AIS lesion found in hysterectomy specimens. Two cases (10.53%) of AIS were confirmed by cervical biopsy and/or ECC, but these patients did not undergo LEEP or CKC, and underwent total hysterectomy directly.

All 71 cases of ADC underwent cervical biopsy and/or ECC. Only 23 cases (32.39%) underwent LEEP or CKC biopsy and 63 cases (88.73%) underwent total hysterectomy. Among the 23 patients who underwent a LEEP or CKC, seven (30.43%) were negative at the cutting edge, and 16 cases (69.57%) were positive at the cutting edge. ADC was still visible after total hysterectomy in one case (14.29%) with negative margins, and no residual ADC lesions were found in five cases (31.25%) having a positive margin after hysterectomy. Forty cases (56.34%) of ADC were confirmed by cervical biopsy and/or ECC without LEEP or CKC, and underwent total hysterectomy directly.

All 135 cases of mixed glandular and squamous lesions underwent cervical biopsy and/or ECC; 82 cases (60.74%) underwent LEEP or CKC biopsy and 98 cases (72.59%) underwent total hysterectomy. Among the 82 LEEP or CKC procedures, 31 (37.80%) yielded samples that were negative for the margins, and 51 cases (62.20%) were positive for the margins. Two cases (6.45%) with negative margins still showed lesions following total hysterectomy, while 13 cases (25.49%) with positive margins did not show residual lesions after hysterectomy. Furthermore the diagnosis was confirmed in 30 cases (22.22%) by cervical biopsy and/or ECC without LEEP or CKC, and these patients underwent the total hysterectomy directly.

In summary, all 225 patients (100%) underwent biopsy and/or ECC; 120 (44.44%) underwent LEEP or CKC biopsy, and 178 cases (79.11%) underwent total hysterectomy. There were 49 cases (40.83%) with negative margins in the LEEP or CKC biopsy, and 71 cases (59.17%) with positive margins. After the total hysterectomy, residual lesions were still visible in five cases (10.20%) with negative margins, while no residual lesions were found in 19 cases (26.76%) with positive margins subsequent to hysterectomy (Tables 6 and 7).

**Table 6:** Pathological tissue biopsy findings

Pathology	biopsy/ECC	CKC/LEEP	Hysterectomy
AIS	19(100%)	15(78.95%)	17(89.47%)
ADC	71(100%)	23(32.39%)	63(88.73%)
MASL	135(100%)	82(60.74%)	98(72.59%)

**Table 7:** Residual lesions at the incision margin after CKC/LEEP surgery and after total hysterectomy

Variables		N	Hysterectomy		
			Absent (%)	Present (%)	*P value*
**CKC/LEEP margins**	Negative	49	45(91.83%)	4(8.16%)	
	Positive	71	18(25.35%)	53(74.65%)	<0.01
**AIS**	Negative	11	10(90.91%)	1(9.09%)	
	Positive	4	0(0.00%)	4(100.00%)	<0.01
**ADC**	Negative	7	6(85.71%)	1(14.29%)	
	Positive	16	5(31.25%)	11(68.75%)	<0.05
**MASL**	Negative	31	29(93.55%)	2(6.45%)	
	Positive	51	13(25.49%)	38(74.51%)	<0.01

## Discussion

In this study, the average age at diagnosis of cervical AIS was approximately 38 years, comparable to the reported average age of 35. In the cervical ADC and mixed adenosquamous lesion (MASL) groups, the average age at diagnosis was approximately 46. The average age of tumor patients with high HPV prevalence, as reported in the scientific literature, is 44.8 years, significantly younger than the average age of patients with low HPV prevalence, with an average HPV prevalence of 49.8 years [5].The average age at diagnosis of cervical AIS was lower than that of cervical ADC and MASL [6]. This finding supports the theory that invasive adenocarcinoma develops as a precursor lesion, taking approximately 7–10 years to develop from in situ lesions, indicating an opportunity for screening and testing before progressing to invasive cancer. The success of Pap test cytology in reducing the incidence rate and mortality of cervical cancer is mainly attributable to the early detection and treatment of SIL.

When HPV infection is detected, particularly HPV18 and HPV16, colposcopy should be performed, even if the cytological results are negative. In agreement with other studies, our data show that the pathogenicity of HPV18 is higher than that of HPV16 [7, 8]. Pirog *et al*. [9] found that HPV16 was the most common cause of infection, with a ratio of 1.6 between HPV16 and HPV18. In this study, recognized non-HPV-related cervical adenocarcinoma types have been excluded, and 17 cases (6.59%) did not have any detectable HPV infection. The specific reasons for this need further research. On the one hand, we consider that it may be due to the limited range of detected HPV types, or to limitations in technical sensitivity variability or degradation during sample storage. On the other hand, it may indicate that HPV has limited involvement in some cervical adenocarcinomas, and if the tumor lasts for a long time, HPVDNA may be lost during the tumor progression process. Epigenetic changes such as methylation, chromosomal abnormalities, and mutations in TP53 and other genes occur in cervical cancer, and there are differences between cervical squamous cell carcinoma and adenocarcinoma [10–13]. There have also been studies indicating the complexity of the association between HPV and adenocarcinoma; with the development and progression of ADC, the driving force of HPV or HPV oncogenes is lost. This hypothesis is supported by the discovery of lower p16 [INK4a] expression. Positive expression in HPV- negative ordinary ADC indicates that there does not seem to be continuous E7 expression/pRB isolation/p16 inactivation [14–16].

Our results also showed that the most common abnormal results in cervical screening were ASC-US and HSIL in the cytological examination. Nearly 1/3 of the cases were often initially diagnosed as NILM based on cytology, and only a few of them have true negative cytological interpretations. The main reason is that the histological lesions are deep, not shallow, and the cytology study is often unable to identify the lesion’s location [6]. Most cytological diagnoses are false negatives due to various reasons, such as failure to locate the lesion site during cytological sampling, failure to achieve positive cells on the slides during the production process, and level of knowledge blind spots among cytologists. Cell pathologists should receive specialized training to enhance their ability to recognize various types of cells. For example, the difficulty in diagnosing AIS lies in the presence of subtle cytological features that are often overlooked or misunderstood as other cervical lesions, such as SIL, endometrial cells, tubal metaplasia, endometriosis, or reactive endometrial cells [17]. The diagnosis of AGC has always been challenging for pathologists and lacks good inter-observer consistency. Thus, additional training of pathologists may represent an area of need that could improve the early diagnosis of cervical lesions.

Similar to previous reports, we also found that more than half of cervical glandular lesions are accompanied by squamous epithelial lesions, which are often the first to be detected [2, 10]. Therefore, cervical cytology screening is more effective for detecting changes in squamous intraepithelial lesions than in glandular epithelial diseases; an estimated 30–60% of glandular epithelial lesions are detected by chance during the follow-up of squamous cell abnormalities [17–19]. Overall, cytology has low sensitivity when screening for glandular diseases, and there is strong support for adding HPV testing to cytology to improve the screening performance.

When testing is positive for HPV18 and/or HPV16, there may be glandular disease present, regardless of the cytological results [5]. The management guidelines for cervical cancer screening abnormalities based on the 2019 ASCCP are risk-based guidelines, and are not dependent on screening results. All initially HPV-positive individuals, regardless of the HPV subtype, are recommended to undergo cytological testing using the same standard [20]. When a patient is diagnosed with AGC or AIS by cytology, regardless of the outcome of HPV testing, immediate colposcopy examination, cervical curettage (ECC), and endometrial curettage should be conducted for those over 35 years old. If AGC- FN is not proven through colposcopy, the HSIL/AIS should also be subjected to diagnostic cervical circumcision should be done forHSIL/AIS.

In summary, the advantages of cytological screening are its high specificity and the ability to detect HPV-negative cases; its disadvantages are its poor sensitivity, high requirements for cytological diagnostic physicians, intense subjectivity and significant results differences among pathologists. The advantages of HPV screening are its high sensitivity and its objective and direct results; the disadvantages are its poor specificity and inability to detect HPV-negative cases. The cytological examination is the initial basis for the initial diagnosis of glandular lesions; cytological examination should be combined with HPV detection to improve the sensitivity to detect glandular abnormalities. It is also necessary to strengthen standardized cytology training on cytology and to develop corresponding quality control standards.

In most cases, especially in mixed adenosquamous lesions, the final diagnosis of cervical adenomatous lesions relies on a combination of HP, Pap test, and/or biopsy/ECC testing. If necessary, LEEP/CKC biopsy may also be performed to thoroughly evaluate the glandular lesions thoroughly [17]. In most cases with negative margins, if conservative treatment is required, it is relatively safe. However, 8.16% of patients still had residual glandular lesions after conservative treatments that were visible in total hysterectomy specimens. Notably, regardless of whether the margins were negative or positive cutting edge, our calculated residual rate of the lesion was 47.5% after LEEP/CKC surgery. This high residual rate highlights the difficulty of using conservative treatment for cervical glandular lesions.

In clinical practice, it can be argued that as long as the patient undergoes colposcopy, it is not important whether the tumor cells are considered squamous or glandular epithelial in origin. However, identifying and reporting atypical glandular cells can guide clinicians to pay special attention to the cervical canal and perform cervical curettage, which may lead to an early diagnosis [21].

Castano et al. showed that screening is inefficient in preventing ADC, but effective in detecting stage IA adenocarcinoma. In the absence of screening, adenocarcinoma is usually diagnosed in stage IB or worse. Therefore, stage IA adenocarcinoma is the most common diagnosis among women who undergo periodic screening. Among these women, there has been sufficient time since the last screening to develop stage IA cancer, but not enough time to develop to stage IB or higher [22]. The impact of screening on adenosquamous carcinoma is similar to that of squamous carcinoma, although more stage IB or more severe adenosquamous carcinomas are detected. This finding suggests that squamous components must be stronger and/or appear earlier in the carcinogenic process to be detected through screening.

When screening for cervical cancer, it is necessary to ensure that the procedures being used provide clinical benefits, and efforts should be made to improve the detection rate, reduce the missed diagnosis rate, and reduce unnecessary bodily damage (such as excessive colposcopy and biopsy), as well as to reduce the excessive psychological burden and anxiety associated with screening, while also considering the cost of health resources.

## Data Availability

The data that support the findings of the study are available from the corresponding author upon reasonable request.
